# Diagnostic outcome of pro bono neurogenetic diagnostic service in Sri Lanka: A wealth creation

**DOI:** 10.1038/s41431-023-01525-3

**Published:** 2024-01-23

**Authors:** Lakmal Gonawala, Nalaka Wijekoon, Darshika Attanayake, Pyara Ratnayake, Darshana Sirisena, Harsha Gunasekara, Athula Dissanayake, Ajantha Keshavaraj, Chandra Mohan, Harry W. M. Steinbusch, Eric P. Hoffman, Ashwin Dalal, K. Ranil D. de Silva

**Affiliations:** 1 Centre for Innovations in Biotechnology and Neuroscience, Faculty of Medical Sciences, University of Sri Jayewardenepura, Nugegoda, 10250 Sri Lanka; 2 Department of Cellular and Translational Neuroscience, School for Mental Health and Neuroscience, Faculty of Health, Medicine & Life Sciences, Maastricht University, 6200 Maastricht, The Netherlands; 3 Lady Ridgeway Hospital for Children, Colombo, Sri Lanka; 4 Colombo North Teaching Hospital, Ragama, Sri Lanka; 5 Sri Jayewardenepura General Hospital, Colombo, Sri Lanka; 6 Teaching Hospital Karapitiya, Galle, Sri Lanka; 7 Teaching Hospital Jaffna, Jaffna, Sri Lanka; 8 Department of Bioengineering, University of Houston, Houston, TX USA; 9 School of Pharmacy and Pharmaceutical Sciences, Binghamton University, New York, USA; 10 Diagnostics Division, Centre for DNA Fingerprinting and Diagnostics, Hyderabad, Telangana India; 11 Institute for Combinatorial Advanced Research and Education (KDU-CARE), General Sir John Kotelawala Defence University, Ratmalana, Sri Lanka

**Keywords:** Genetics of the nervous system, Genetic testing

## Abstract

The inherited disease community in Sri Lanka has been widely neglected. This article aimed to present accumulated knowledge in establishing a pro bono cost-effective national, island-wide, free-of-charge molecular diagnostic service, suggesting a model for other developing countries. The project provided 637 molecular diagnostic tests and reports free of charge to a nation with limited resources. We pioneered the implementation of mobile clinics and home visits, where the research team acted as barefoot doctors with the concept of the doctor and the researcher at the patient’s doorstep. Establishing pro bono, cost-effective molecular diagnostics is feasible in developing countries with limited resources and state funding through the effort of dedicated postgraduate students. This service could provide an accurate molecular diagnosis of Duchenne muscular dystrophy, Huntington’s disease, Spinocerebellar ataxia, and Spinal muscular atrophy, a diagnostic yield of 54% (343/637), of which 43% (147/343) of the patients identified as amenable for available gene therapies. Initiated human resource development by double doctoral degree opportunities with international collaborations. Established a neurobiobank and a national registry in Sri Lanka, a rich and unique repository, wealth creation for translational collaborative research and sharing of information in neurological diseases, as well as a lodestar for aspiring initiatives from other developing countries.

## Introduction

The inherited diseases community in Sri Lanka has been highly neglected, often being labeled as “incurable”. It is noteworthy that comparatively little attention seems to be given to patients with neurogenetic diseases. Neurogenetic testing is almost nonexistent in Sri Lankan government hospitals and is only available in a few private sector centers at exorbitant costs. Moreover, research in developing countries has been hampered by limited advanced clinical resources (lack of clinicians/experts having sound knowledge and experience in clinical genetics and lack of standardized clinical guidelines and specific clinical infrastructure) and far-to-approach sporadically localized genetic service centers [1–3]. This has resulted in needy patients undergoing unavoidable clinical investigations, leading to multiple indefinite diagnoses [4, 5].

Addressing these issues, the corresponding author pioneered in establishing a free-of-charge neuromolecular diagnostic service at a governmental institute, despite many challenges, efforts led to the successful molecular diagnosis of 343 out of 637 patients with rare diseases, representing a 54% confirmation rate as summarized in Table 1. We assess the demographic features, diagnostic yield through conventional PCR and Multiplex Ligation Dependent Probe Amplification (MLPA) analysis, a pro bono neurogenetic diagnostic service in national single center in Sri Lanka.

**Table 1:** Diagnostic outcome and baseline characteristics of patients with, Neuromuscular Disorders and Polyglutamine (polyQ) diseases identified through Pro bono neurogenetic diagnostic service.

Neuromuscular disorders				
Patients with the characteristic clinical findings suspected for	**Myopathy (*n*** = **248)**			**SMA (*n*** = **66)**
	**DMD**	**BMD**	LGMD*	
Number of Genetically confirmed cases	138	11	03	22
Tested mutations not detected	89		07	44
Associated Gene/ Genes (MIM#)	DMD (300377)	DMD (300377)	CAPN3 (114240)	SMN1 (600354)
Mutation detection Method	MLPA	MLPA	WES*	MLPA
Mutation Identified (%)	Deletions- 125/138 (90%) Duplications-13/138 (9.4%)	Deletions-11 (100%) Duplications-NA	Missense CAPN3-Exon 10 c.1342C > T (All 3 patients) Missense GNE c.2179G > A (Data available for 2 patients)	Based on SMN2 copy number, SMA type 0- 4 SMA type 1- 4 SMA type 2- 7 SMA type 3- 6 SMA type 4- 1
Cases with Family History	21/138 (15%)	4/11 (36%)	All 03 are siblings	1/22 (5%)
Cases with Consanguinity	8/138 (6%)	NA	3/3 (100%)	5/22 (23%)
Age range (Mean)	1.5–18 Yrs (8 Yrs)	12–37 Yrs (21 Yrs)	22–34 Yrs (26 Yrs)	SMA Type 0- 10-180 days (73 days) SMA Type 1- 2 - 36 months (24 months) SMA Type 2-1-8 Yrs (5 Yrs) SMA Type 3-5-43 Yrs (18 Yrs) SMA Type 4-42 Yrs (NA)
Age of onset range (Mean)	1–8 Yrs (4 Yrs)	11–15 Yrs (13 Yrs)	Sibling 01- 18 Yrs Sibling 02- 12 Yrs Sibling 03- 10 Yrs	SMA Type 0-1-60 days (30 days) SMA Type 1-2-12 months (5 months) SMA Type 2-6-24 months (15 months) SMA Type 3-3-18 Yrs (8 Yrs) SMA Type 4-39 Yrs (NA)
Gender (M/F)	138/0	11 /0	02/01	13/09
NSAA mean	14	21	Sibling 01- Wheelchair bound Sibling 02- 27 Sibling 03- 12	NA
Hammersmith Scale Mean	NA	NA	NA	SMA Type 0- NA SMA Type 1- NA SMA Type 2- 33 SMA Type 3- 33 SMA Type 4- NA
Mean CPK	15143 U/L	4000 U/L	NA	NA
Unique Cases/ Remarks	Identical Twins with DMD (7th Reported Case Worldwide, genetically conformed sporadic case.	–	3 members with the same family diagnosed with limb girdle 2 A with three generation of maternal and paternal consanguinity	–
Trinucleotide repeat Disorders				
Patients with the characteristic clinical findings suspected for	**SCA (*n*** = **236)**			**HD (n-87)**
Number of Genetically confirmed cases	123			45
Tested mutations not detected	113			42
Mutation detection Method	Single plex PCR			Single plex PCR
Mutation Identified	SCA type 1-61	SCA type 2-23	SCA type 3-39	N/A
Cases with Family History	32	03	07	09
Mean Age	40.5	41.6	46.0	47.8
Mean Age of onset range	36.0	35.9	41.3	43.5
Gender (F/M)	35/26	9/14	16/23	21/24
Mean score of Scale for the Assessment and Rating of Ataxia (SARA)	16.3	14.5	12.1	N/A
Mean score of UHDRS Motor	N/A	N/A	N/A	40.0 ± 12
Mean score of UHDRS Functional Capacity	14.5	18.8	17.8	17.4
Mean score of UHDRS Behavioral	N/A	N/A	N/A	33.7 ± 13
Mean score of Addenbrooke’s Cognitive Examination	34.7	37.3	46.5	49.8
Unique Cases/ Remarks	Localization of 25 SCA type 1 genetically confirmed patients with a common ancestry in three villages			

^*^All LGMD patients tested negative for MLPA, and three patients from the same family underwent whole exome sequencing.

In Low Middle-Income Countries, a critical shortage of health-care workers such as medical geneticists and scientists is clearly evident due to the movement of skilled labor to developed countries [6]. Thereby, in literature to the best of our knowledge pro bono molecular genetic testing services were reported only in India (http://gomed.igib.in) and the West Indies [7]. In this context this article aimed to present accumulated knowledge in establishing a pro bono cost-effective national, island-wide, free-of-charge molecular diagnostic service, suggesting a model for other developing countries. It shows the diagnostic yield of selected rare neuromuscular, and trinucleotide repeat disorders in Sri Lanka, which were executed with minimal resources and cost, the uphill task of establishing a neurobiobank, and how investing in human resources is crucial for sustainability.

## Materials and Methods

**Fig. 1:**
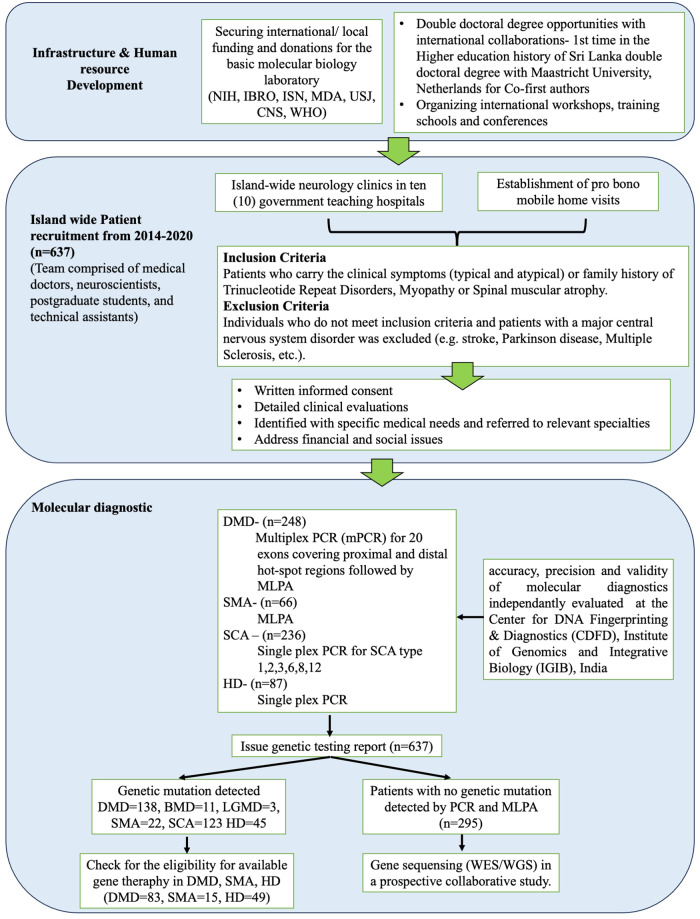
Summery of each key strategic areas in establishing of pro bono molecular diagnostic service. The key strategic areas comprised of Infrastructure and human resource development achieved through international/ local funding and Double doctoral degree opportunities with international collaborations, Island wide Patient recruitment based on neurology clinic visits and pro bono mobile home visits followed by Molecular diagnostics.

The establishment of pro bono molecular diagnostic model was based on following key strategic areas (Fig. 1),

### Infrastructure development

Securing international/ local funding and donations for the basic molecular biology laboratory infrastructure development and human resource development

The corresponding author received a donation of equipment from the National Institute of Neurological Disorders and Stroke (NINDS) of the National Institute of Health (NIH), USA in 2006 through the International Brain Research Organization Asia Pacific Regional Committee (IBRO-APRC). Additionally, equipment was donated by the Chinese Neuroscience Society (CNS) in 2014. This was a pivotal stepping stone for the corresponding author as the principal investigator (PI) in setting up the pro bono molecular diagnostic service at the Interdisciplinary Center for Innovation in Biotechnology and Neuroscience, University of Sri Jayewardenepura (USJ), Sri Lanka. As of 2021, this service is being supported by the General Sir John Kotelawala Defence University in Sri Lanka. The World Health Organization (WHO) in 2012 and the Muscular Dystrophy Association (MDA) in the USA in 2010 funded the project to ensure the sustainability of this approach. Research grants were received from the University of Sri Jayewardenepura, General Sir John Kotelawala Defence University, and Ministry of Primary Industries, Sri Lanka.

### Patient recruitment

Island-wide pro bono mobile clinics in selected government hospitals and home visits in the Western, North-Western, North Central, Central, Southern Provinces and Northern Provinces. In the Northern Province, the Jaffna Teaching Hospital was the hub for patient recruitment. It is noteworthy that offering such medical genetic services to patients from Jaffna has been hindered for nearly 30 years because of the civil war.

Patient recruitment was based on island-wide neurology clinics in ten (10) government teaching hospitals from 2014–2020. Independent registries were maintained by the pro bono team and representative doctors at each neurology clinic.

### Establishment of pro bono mobile home visits

During clinic visits, the visiting pro bono team observed that patient participation in the clinic for follow-up studies was hindered. This was due to:

I. Patients’ hopelessness mindset, which was developed due to the labeling of these patients as “incurable” in the initial clinic visit and lack of proper counseling.
II. High cost of travel to the hospital for disabled or wheelchair-bound patients.
III. The poor mutual understanding and relationship between patients and the healthcare team in the clinic, creating a gap that made the patients and their family members reluctant to express their true experience of social, emotional, and financial hardships of living with an inherited disease.

This led to the establishment of pro bono mobile home visits by the visiting pro bono team, who acted as barefoot doctors with the concept of the doctor and the researcher at the patient’s doorstep.

The team comprised medical doctors, neuroscientists, postgraduate students, and technical assistants. Detailed clinical evaluations were performed, and the patients were directed to the nearest neurology clinic at a government hospital for further medical assistance. Moreover, frequent home visits helped develop a rapport with the pro bono team and the patient and their family members, which was beneficial in identifying medical, social, and financial problems. Patients who were identified with specific medical needs (psychiatric, cardiology, occupational therapy, physiotherapy, and counseling) were referred to relevant specialties. Financial and social issues were addressed and facilitated through relevant government departments, and progress was followed by the pro bono research team.

This approach met the ethical guidelines of the Sri Lankan Institutional Review Board and complied with the Declaration of Helsinki. Written informed consent was obtained from each proband where applicable. Informed consent was obtained from a proxy if the patient was unable to provide consent on their own.

### Molecular diagnostic

This study utilized the molecular diagnostic approach established in-house following published papers as described in Wijekoon et al. 2023 [8]. A summary of this approach is as follows. The initial diagnostic test for detecting deletions and duplications followed a level one testing approach, utilizing Multiplex PCR (mPCR) for 20 exons covering proximal and distal hot-spot regions of the Duchenne Muscular Dystrophy (*DMD)* gene as described by Chamberlain et al. [9] and Beggs et al. [10] followed by the MLPA assay (MRC Holland SALSA MLPA Probe mixes P034 and P035) for all the clinically diagnosed dystrophinopathy patients. The diagnostic procedure was established utilizing the primary molecular diagnostic recommendations as outlined by Abbs et al. in 2010, as well as the revised edition by Fratter et al. in 2020, in alignment with the European Molecular Quality Genetics Network’s (EMQN) optimal practice guidelines for genetic testing in dystrophinopathies [11, 12]. In order to ascertain the impact of variations on the reading frame, the frame-shift checker available on the Leiden Muscular Dystrophy website (www.dmd.nl) was utilized to scrutinize all identified deletions and duplications.

Spinocerebellar Ataxia (SCA) genetic analysis was also based on the EMQN guidelines [13] where we have modified SCA testing priorities and considered testing for further genes on a case-by-case basis on the basis of local population disease frequencies, the geographic and ethnic origin of the patient and the information provided in the referral with regard to clinical presentation and the apparent mode of inheritance. Huntington’s Disease (HD) genetic analysis was also based on the EMQN guidelines [14].

This was funded by foreign and local donations and research grants to the PI, with inadequate support from the government, which was challenging. Molecular diagnostics and genomic data interpretation were performed by two PhD scholars in neuroscience (LG and NW, the co-primary authors of this manuscript).

The accuracy, precision and validity of molecular diagnostics performed in our laboratory were confirmed by re-evaluating random samples of DMD, HD, SCA and SMA for genetic mutations following the same protocol at collaborative laboratories in India (Center for DNA Fingerprinting & Diagnostics, Institute of Genomics and Integrative Biology). Moreover, at the inception, the positive controls were provided by the Center for DNA Fingerprinting & Diagnostics, Institute of Genomics and Integrative Biology, India. The interpretation of genetic testing reports was based on the guidelines published in NCBI Gene reviews [15–20].

### Human resource development

Human resource development by double doctoral degree opportunities with international collaborations and disseminating scientific knowledge by organizing international workshops, training schools and conferences.

## Results

### Molecular Diagnostics

A nationwide, free-of-charge, traditional molecular diagnostic service was offered to utilize basic equipment (i.e., a polymerase chain reaction [PCR] machine and gel documentation system). The project provided 637 molecular diagnostic tests (PCR-based methods) and reports free of charge as a service to the nation (Table 1).

With a diagnostic yield of 54% (343/637), our approach may accurately diagnose DMD, HD, SCA, and SMA patients using molecular means. Of the myopathy patients in this cohort, we could give molecular diagnostic for 55% (131/236). It’s interesting to note that 96% (131/136) of the patients in this cohort with deletion mutations could be diagnosed by mPCR. A group of patients with SCA type 1 in three villages in the southern part of Sri Lanka was identified (*n* = 29/61). 41 patients (DMD, 9; SMA, 2; SCA, 17; and HD, 13) in our cohort had afflicted siblings or relatives. Of the 343 genetically diagnosed patients, 147 (DMD, 83; SMA, 15; and HD, 49) qualified for gene therapy.

### Cost effectiveness

The authors were successful in providing a low-cost molecular diagnostic approach, including the multiplex ligation probe amplification test and PCR-based molecular diagnostics, with 27–54 USD spent on the chemicals and consumables per test. The cost inclusive of utility and labor is 42-69 USD. However, it is noteworthy that the costs associated with labor and utilities were covered by the institute. This cost was cheaper than the costs reported in the literature for regional countries (inclusive of utility and labor); in Pakistan, the cost of a PCR test for 22 exons of the DMD gene is approximately 333 USD [21], and in India, the same test costs approximately 52 USD [22]. Moreover, in India, genetic testing for HD and SCA panels (types 1, 2, and 3) costs 35 USD and 79 USD, respectively.

### Benefit to the patient

With our efforts, the patients and their families benefited from a definitive diagnosis achieved through a free of charge molecular diagnostic report, resulting in better disease management and allowing them to understand the implications. The patients were referred for formal physical, speech, nutritional, and rehabilitative therapy and counseling under the supervision of the respective consultants. Moreover, patient’s specific medical needs and financial and social issues were addressed by referring to relevant medical specialties and relevant government departments.

### Human resource development

Testing genetic diseases, conducting research, and training personnel who can make genetic testing more widely available to the public and serve the needs of the inherited disease community in Sri Lanka are crucial. In this regard, the author successfully initiated:

- Double doctorate opportunities between Maastricht University in the Netherlands and Sri Lanka; the first double doctorate was awarded in 2020, with the corresponding author as the PI. Two more PhDs are registered for double doctorate opportunities with Maastricht University, which will result in two neuroscientists with international training capable of continuing molecular diagnostic services.
- Six international neuroscience and molecular biology workshops and postgraduate training through exchange programs with support from the IBRO and the International Society for Neurochemistry. The Center for DNA Fingerprinting and Diagnostics, India; CSIR Institute of Genomics and Integrative Biology, India; Alzheimer’s Association, USA; The Movement Disorders Society; and the National Institute of Advanced Industrial Science and Technology, Japan provided training.

More than 15 Medical Graduates were recruited for this ongoing research during the study period and were trained in neurogenetics and clinical neuroscience.

## Discussion

To the best of our knowledge, our service stands out due to its unique approach of providing doctors and researchers at the doorstep, in contrast to the reported pro-bono molecular genetic testing services in India and the West Indies.

A definitive diagnosis beneficially resulted in the patients, their families, the community, and the inherited disease community as follows:

- Cost-effective disease management invalidates unnecessary empirically expensive therapies that could have serious side effects; prevents comorbidities; reduces morbidity, mortality, and secondary manifestations; and improves the quality of life and mental well-being.
- Neurologists are guided in understanding some of their most challenging patients who would have been left without answers and entangled in a diagnostic odyssey. Two patients in whom the physician initially suspected limb girdle muscular dystrophy (LGMD) and Pompe disease were later confirmed to have DMD via genetic testing.
- Potential prevention through genetic counseling and multidisciplinary treatment increases quality of life and life expectancy. An optimal treatment plan can promote prevention and early detection, allowing family planning. In our cohort, 41 cases (DMD, 9; SMA, 2; SCA, 17; and HD, 13), had affected siblings or family members. This indicates the need to establish carrier detection [23] and preimplantation genetic testing [24] within a proper legal framework, along with genetic and reproductive counseling, which is far from the current reality in Sri Lanka.
- Enrollment in clinical trial registries: 147 out of 343 (DMD,83; SMA,15; and HD, 49) patients were eligible for gene therapy [8, 25]. Fruitful discussions are underway with the Working Group consist of multiple stakeholders with expertise in the advocacy, diagnosis, treatment of rare neuromuscular disorders. The members include clinical experts from Sydney Children’s Hospital, Australia working in partnership with international patient advocate group, Advocacy Beyond Borders and diagnostic and therapeutic pharmaceutical stakeholders. The Committee members are self-funded with no financial or commercial conflicts of interest.
- Creation of National Wealth: established a neuro-biobank [26]: seventy six (76) aging human autopsy brains [27] and a DNA/serum repository of 2700 neurological disease patients: stroke (n-1200), Parkinson’s (n-350), neurodegenerative, neuromuscular, rare disease, and 500 controls. The rare disease cohort (Table 1) will be an asset for linking East and West, as 287 (46%) of the rare-disease cohort were negative for common mutations and a sporadic case of monozygotic twins genetically confirmed with DMD was identified. International collaborations to identify novel mutations and perform epigenetic analyses. Development of genetic networks in innovative multicenter translation therapeutic projects.
- Further cost reduction is possible by narrowing the number of SCA tests performed per patient based on geographical localization, which was based on an identified cluster (*n* = 29/61) of patients with SCA type 1 in three villages in the southern region of Sri Lanka.
- In our study multiplex PCR(mPCR) could provide molecular diagnosis for 55% (131/236) of the myopathy patients, Intriguingly 96% (131/136) of the patients with deletion mutations could be diagnosed by mPCR. Thus utilization of mPCR as an initial molecular diagnostic method is deemed cost-effective for nations with restricted resources, given its 96% rate of detection for deletions.
- Using serum samples of DMD patients from the biobank, Wijekoon et al. (2023) conducted a study correlating serum protein signatures with cognitive performance in DMD patients. Intriguingly, the study’s novel findings suggested a common pathogenic mechanism underlying for both Alzheimer’s disease and DMD cognitive impairment [27–29]. Further more promising fluid biomarkers for SCAs have not yet been validated, the authors were able to identify and validate novel serum biomarkers in SCA (Unpublished data).

Sri Lanka is served by 44 neurologists (1 neurologist per 500,000 people), reflecting the gross mismatch between the burden of neurological disorders and the availability of resources [30]. These numbers are much worse when the Indian subcontinent is considered; in India and Pakistan, there is one neurologist per one million people. This reflects a shortage of neurologists in regional countries [31, 32]. Patients affected by neurogenetic diseases in Sri Lanka must travel between 40 and 50 km to the closest regional hospital to seek specialty care. This results in the high cost of travel to hospitals for disabled or wheelchair-bound patients, which is greater than the monthly income of most families [33].

Most of Sri Lanka’s population (81.5%) live in rural areas [34]. A recent study identified that 42% (9.6 million people) lives below the poverty line [35]. Notably, the majority (80.9%) of the poor live in rural areas that are underdeveloped in healthcare, public transportation, and education. This has resulted in disparities in access to healthcare between rural and urban populations, mainly due to the lack of retention of healthcare workers in rural areas. This disparity has significantly affected the estate sector, as reflected by poorer health outcomes than in the urban and rural sectors in Sri Lanka. In line with this, it has been reported that maternal and child Health and nutritional and psychosocial indicators lag behind in the estate sector [36]. Moreover, the Multidimensional Poverty Index (MPI) of children reveals that 42.2% of children 0–4 y of age are poor, and one-third of young children are undernourished [37].

### The key findings, limitations and future directions

The key findings of this study are as follows: (i) The hindrance of patient participation in follow-up studies at the clinic can be effectively addressed by implementing mobile home visits. (ii) It is possible to achieve a diagnostic yield of 54% for patients with DMD, HD, SCA, and SMA, even in settings with minimal resources. (iii) Cost reduction can be achieved by utilizing mPCR as an initial molecular diagnostic method for DMD, as it has a 96% detection rate for deletions and by narrowing the number of SCA tests performed per patient based on their geographical localization.

The clinical evaluation conducted during mobile clinics at patients’ homes may be influenced by the infrastructure available at their residences. In such cases, it may be necessary to arrange for the patient’s transportation to the nearest hospital when deemed necessary. Furthermore, in Sri Lanka, it is observed that individuals affected by progressive neuro-degenerative genetic diseases often conceal their condition due to cultural and social factors. This is primarily driven by the stigma associated with these diseases. Consequently, once their condition becomes known, these patients are often isolated and excluded from society. Therefore, as a result of these factors, patients often experience underdiagnosis. Thirdly, the lack of a comprehensive patient recording system in many government hospitals for rare neurological diseases, coupled with the absence of a permanent solution, has resulted in a significant number of patients not being able to attend regular clinics. Consequently, it becomes challenging to provide adequate coverage for all patients.

**Fig. 2:**
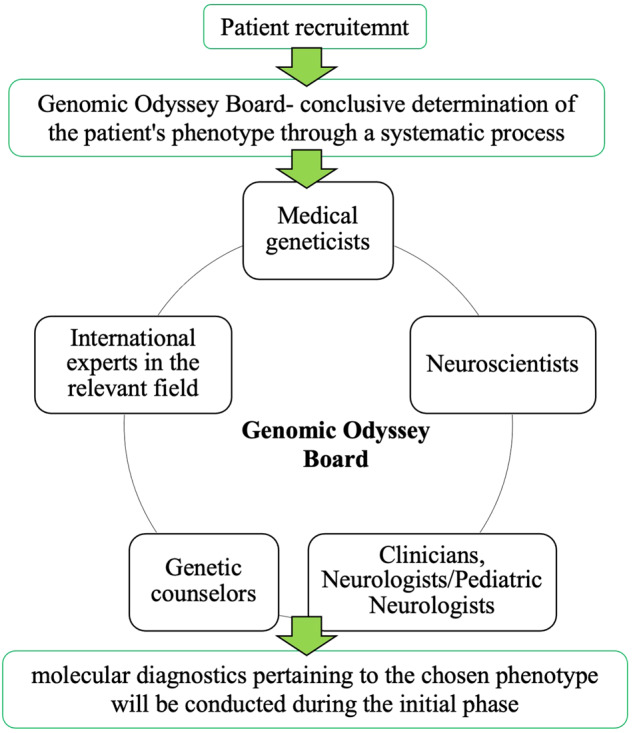
The proposed structure for “Genomic Odyssey Board”. It is proposed to have multidisciplinary panel of experts including medical geneticists, Neuroscientists, Clinicians, Neurologists/ pediatric Neurologists, Genetic counselors and international experts in the relevant field. This approach will lead to molecular diagnostics pertaining to the chosen phenotype.

Although the current detection rate of our approach stands at 55%, there is potential for improvement through the implementation of a phenotype-based prioritization strategy for candidate genes. In this particular scenario, it is recommended to refine the initial clinical diagnosis of the patient by convening a “Genomic Odyssey Board”. (Fig. 2) This board will consist of esteemed professionals including Medical Geneticists, Neuroscientists, Clinicians, Neurologists/Pediatric Neurologists, Genetic Counselors, and International experts in the relevant field. The primary objective of this board is to reach a conclusive determination of the patient’s phenotype through a systematic process. Subsequently, the molecular diagnostics pertaining to the chosen phenotype will be conducted during the initial phase.

In conclusion, we show that establishing pro bono cost-effective molecular diagnostics is feasible in developing countries with limited resources and state funding with dedicated postgraduate students. Moreover, we show that accurate molecular diagnosis of DMD, HD, SCA, and SMA patients, a diagnostic yield of 54% (343/637), of which 43% (147/343) of the patients identified as amenable for available gene therapies using basic equipment, which can serve as a model for other developing countries, benefiting affected individuals with a definitive molecular diagnosis, their families, the health care systems and the community at large by genetic counseling and prevention. The work has resulted in establishing neurobiobank and a national registry in Sri Lanka, a rich and unique repository, a wealth creation for translational collaborative research and sharing of information in neurological diseases, as well as a lodestar for aspiring initiatives from other developing countries with limited resources.

## Data Availability

The datasets used and/or analysed during the current study are available from the corresponding author on reasonable request.

## References

[1] Pradhan S, Sengupta M, Dutta A, Bhattacharyya K, Bag SK, Dutta C, et al. Indian genetic disease database. Nucleic acids Res. 2010;39:D933–D8. 21037256 10.1093/nar/gkq1025 PMC3013653

[2] Aggarwal S, Phadke SR. Medical genetics and genomic medicine in India: current status and opportunities ahead. Mol Genet Genom Med. 2015;3:160. 10.1002/mgg3.150 PMC4444157 26029702

[3] Kasthuri A. Challenges to healthcare in India-The five A’s. Indian J Commun Med: Off Publ Indian Assoc Prev Soc Med 2018;43:141. 10.4103/ijcm.IJCM_194_18 PMC6166510 30294075

[4] Knight AW, Senior TP. The common problem of rare disease in general practice. Med J Aust. 2006;185:82–3. 16842062 10.5694/j.1326-5377.2006.tb00477.x

[5] Zurynski Y, Frith K, Leonard H, Elliott E. Rare childhood diseases: how should we respond? Arch Dis Child. 2008;93:1071–4. 18684747 10.1136/adc.2007.134940

[6] Thong M-K, See-Toh Y, Hassan J, Ali J. Medical genetics in developing countries in the Asia-Pacific region: challenges and opportunities. Genet Med. 2018;20:1114–21. 30093710 10.1038/s41436-018-0135-0

[7] Sobering AK, Li D, Beighley JS, Carey JC, Donald T, Elsea SH, et al. editors. Experiences with offering pro bono medical genetics services in the West Indies: Benefits to patients, physicians, and the community. Am J Med Genet C: Semin Med Genet; Wiley Online Library (2020). 10.1002/ajmg.c.31871 PMC8683562 33274544

[8] Wijekoon N, Gonawala L, Ratnayake P, Sirisena D, Gunasekara H, Dissanayake A, et al. Gene therapy for selected neuromuscular and trinucleotide repeat disorders–An insight to subsume South Asia for multicenter clinical trials. IBRO Neurosci Rep. 2023;14:146–53. 36819775 10.1016/j.ibneur.2023.01.009 PMC9931913

[9] Chamberlain JS, Gibbs RA, Rainer JE, Nguyen PN, Thomas C. Deletion screening of the Duchenne muscular dystrophy locus via multiplex DNA amplification. Nucleic acids Res. 1988;16:11141–56. 3205741 10.1093/nar/16.23.11141 PMC339001

[10] Beggs AH, Koenig M, Boyce FM, Kunkel LM. Detection of 98% of DMD/BMD gene deletions by polymerase chain reaction. Hum Genet. 1990;86:45–8. 2253937 10.1007/BF00205170

[11] Fratter C, Dalgleish R, Allen SK, Santos R, Abbs S, Tuffery-Giraud S, et al. EMQN best practice guidelines for genetic testing in dystrophinopathies. Eur J Hum Genet. 2020;28:1141–59. 32424326 10.1038/s41431-020-0643-7 PMC7608854

[12] Abbs S, Tuffery-Giraud S, Bakker E, Ferlini A, Sejersen T, Mueller CR. Best practice guidelines on molecular diagnostics in Duchenne/Becker muscular dystrophies. Neuromuscul Disord. 2010;20:422–7. 20466545 10.1016/j.nmd.2010.04.005

[13] Sequeiros J, Martindale J, Seneca S. EMQN Best Practice Guidelines for molecular genetic testing of SCAs. Eur J Hum Genet. 2010;18:1173–6. 20179742 10.1038/ejhg.2010.8 PMC2987475

[14] Losekoot M, Van Belzen MJ, Seneca S, Bauer P, Stenhouse SA, Barton DE. EMQN/CMGS best practice guidelines for the molecular genetic testing of Huntington disease. Eur J Hum Genet. 2013;21:480–6. 22990145 10.1038/ejhg.2012.200 PMC3641377

[15] Darras BT, Menache-Starobinski CC, Hinton V, Kunkel LM. Dystrophinopathies. In: Darras BT, Jones HR Jr, Ryan MM, De Vivo DC, editors. Neuromuscular Disorders of Infancy, Childhood and Adolescence: A Clinician’s Approach. 2 ed. San Diego, CA: Academic Press; 2015, p. 551–92.

[16] Angelini C. Calpainopathy. 2005 May 10 [Updated 2022 Dec 1]. In: Adam MP, Feldman J, Mirzaa GM, et al., editors. GeneReviews® [Internet]. Seattle (WA): University of Washington, Seattle; 1993–2023. Available from: https://www.ncbi.nlm.nih.gov/books/NBK1313/. 20301490

[17] Prior TW, Leach ME, Finanger E. Spinal Muscular Atrophy. 2000 Feb 24 [Updated 2020 Dec 3]. In: Adam MP, Feldman J, Mirzaa GM, et al., editors. GeneReviews® [Internet]. Seattle (WA): University of Washington, Seattle; 1993–2023. Available from: https://www.ncbi.nlm.nih.gov/books/NBK1352/. 20301526

[18] Caron NS, Wright GEB, Hayden MR. Huntington Disease. 1998 Oct 23 [Updated 2020 Jun 11]. In: Adam MP, Feldman J, Mirzaa GM, et al., editors. GeneReviews® [Internet]. Seattle (WA): University of Washington, Seattle; 1993–2023. Available from: https://www.ncbi.nlm.nih.gov/books/NBK1305/. 20301482

[19] Aoki M, Takahashi T. Dysferlinopathy. 2004 Feb 5 [Updated 2021 May 27]. In: Adam MP, Feldman J, Mirzaa GM, et al., editors. GeneReviews® [Internet]. Seattle (WA): University of Washington, Seattle; 1993–2023. Available from: https://www.ncbi.nlm.nih.gov/books/NBK1303/. 20301480

[20] Perlman S. Hereditary Ataxia Overview. 1998 Oct 28 [Updated 2023 Nov 16]. In: Adam MP, Feldman J, Mirzaa GM, et al., editors. GeneReviews® [Internet]. Seattle (WA): University of Washington, Seattle; 1993–2023. Available from: https://www.ncbi.nlm.nih.gov/books/NBK1138/. 20301317

[21] Naseem N, Jawad U. Enabling the disabled: Call for intercepting disability surge in Pakistan. J Fam Med Prim Care. 2017;6:721. 10.4103/jfmpc.jfmpc_50_17 PMC5848386 29564251

[22] Srivastava P, Malhotra KP, Husain N, Malhotra HS, Kulshreshtha D, Anand A. Diagnosing muscular dystrophies: comparison of techniques and their cost effectiveness: a multi-institutional study. J Neurosci Rural Pract. 2020;11:420–9. 32753807 10.1055/s-0040-1713301 PMC7394627

[23] Laing NG, Ong RW, Ravenscroft G. Genetic neuromuscular disorders: what is the best that we can do? Neuromuscul Disord. 2021;31:1081–9. 34736628 10.1016/j.nmd.2021.07.007

[24] Blancato JK, Wolfe E, Sacks PC. Preimplantation genetics and other reproductive options in Huntington disease. Handb Clin Neurol. 2017;144:107–11. 28947109 10.1016/B978-0-12-801893-4.00009-2 PMC5837037

[25] Farrar MA, Calotes-Castillo L, De Silva R, Barclay P, Attwood L, Cini J, et al. Gene therapy-based strategies for spinal muscular atrophy—an Asia-Pacific perspective. Mol Cell Pediatr. 2023;10:1–11. 37964159 10.1186/s40348-023-00171-5 PMC10645685

[26] Wijekoon N, Gonawala L, Wijesinghe P, Steinbusch HW, Mohan C, de Silva KRD. A biobank in Sri Lanka that links East and West. Lancet Neurol. 2020;19:972. 33212058 10.1016/S1474-4422(20)30405-1

[27] Wijesinghe P, Shankar S, Yasha T, Gorrie C, Amaratunga D, Hulathduwa S, et al. Vascular contributions in Alzheimer’s disease-related neuropathological changes: First autopsy evidence from a South Asian aging population. J Alzheimer’s Dis. 2016;54:1607–18. 27589527 10.3233/JAD-160425

[28] Wijekoon N, Gonawala L, Ratnayake P, Dissanayaka P, Gunarathne I, Amaratunga D, et al. Integrated genomic, proteomic and cognitive assessment in Duchenne Muscular Dystrophy suggest astrocyte centric pathology. Heliyon. 2023;9:e18530. 10.1016/j.heliyon.2023.e18530 PMC10432191 37593636

[29] Wijekoon N, Gonawala L, Ratnayake P, Amaratunga D, Hathout Y, Mohan C, et al. Duchenne muscular dystrophy from brain to muscle: the role of brain dystrophin isoforms in motor functions. J Clin Med. 2023;12:5637. 37685704 10.3390/jcm12175637 PMC10488491

[30] LANKA, M.O.H.S. Annual Health Bulletin 2020; Ministry of Health, Sri Lanka: Colombo, Sri Lanka, 2020. https://www.health.gov.lk/wp-content/uploads/2023/09/Annual-Health-Bulletin-2020Updated-on-2023.02.02.pdf.

[31] Farooq A, Venketasubramanian N, Wasay M. Stroke Care in Pakistan. Cerebrovasc Dis Extra. 2021;11:118–21. 10.1159/000519554 PMC8613565 34695824

[32] Neurology WFo. The Future of Neurology in India 2020 [Available from: https://worldneurologyonline.com/article/the-future-of-neurology-in-india/.

[33] Samaranayake N, Dissanayaka P, Gunarathna I, Gonawala L, Wijekoon N, Rathnayake P, et al. What we fail to see in neuro-genetic diseases: a bird’s eye view from the developing world. Ann Neurosci. 2020;27:91–7. 34556946 10.1177/0972753120950069 PMC8454996

[34] Census Do, Statistics. Household income and expenditure survey 2012/13. Colombo, Sri Lanka: Child Youth and Cultural Affairs Sri Lanka; 2015. http://www.statistics.gov.lk/Resource/en/IncomeAndExpenditure/HouseholdIncomeandExpenditureSurvey2012-2013FinalReport.pdf.

[35] FT D. Nearly 10 m Sri Lankans affected by poverty: Study: Daily FT; 2022 [Available from: https://www.ft.lk/front-page/Nearly-10-m-Sri-Lankans-affected-by-poverty-Study/44-740898.

[36] Department of Census and Statistics SL. Household income and expenditure survey 2016. Colombo: Ministry of National Policies and Economic Affairs. (2018).

[37] Department of Census and Statistics SL. Multidimensional Poverty in Sri Lanka. (2019).

